# Use of Silica Based Materials as Modulators of the Lipase Catalyzed Hydrolysis of Fats under Simulated Duodenal Conditions

**DOI:** 10.3390/nano10101927

**Published:** 2020-09-27

**Authors:** Sara Muñoz-Pina, Pedro Amorós, Jamal El Haskouri, Ana Andrés, José V. Ros-Lis

**Affiliations:** 1 Inorganic Chemistry Department, REDOLí Group, Universitat de València, Burjassot, 46100 Valencia, Spain; sara.munoz@uv.es; 2 Instituto de Ciencia de Materiales, Universitat de València, C/Catedrático José Beltrán 2, 46980 Paterna Valencia, Spain; pedro.amoros@uv.es (P.A.); Jamal.Haskouri@uv.es (J.E.H.); 3 Instituto Universitario de Ingeniería de Alimentos para el Desarrollo (IUIAD-UPV), Universitat Politècnica de València Camino de Vera s/n, 46022 Valencia, Spain; aandres@upv.es

**Keywords:** lipase, mesoporous silica nanoparticle, Stöber, UVM-7, fat hydrolysis, Partial Least Square Regression

## Abstract

The effect of silica materials and their functionalization in the lipase catalyzed fat hydrolysis has been scarcely studied. Fifteen silica materials were prepared and their effect on the fat hydrolysis was measured, under simulated duodenal conditions, using the pH-stat method. The materials are composed of the combination of three supports (Stöber massive silica nanoparticles, Stöber mesoporous nanoparticles and UVM-7) and four surface functionalizations (methyl, trimethyl, propyl and octyl). In addition, the non-functionalized materials were tested. The functional groups were selected to offer a hydrophobic character to the material improving the interaction with the fat globules and the lipase. The materials are able to modulate the lipase activity and their effect depending on the support topology and the organic covering, being able to increase or reduce the fat hydrolysis. Depending of the material, relative fat hydrolysis rates of 75 to 140% in comparison with absence of the material were obtained. The results were analyzed by Partial Least Square Regression and suggest that the alkyl modified mesopores are able to improve the fat hydrolysis, by contrast the non-porous nanoparticles and the textural pores tend to induce inhibition. The effects are more pronounced for materials containing long alkyl chains and/or in absence of taurodeoxycholate.

## 1. Introduction

Digestion is a complex action involving chemical, physical and biological processes [1]. Most of the hydrolysis of fats and their absorption occurs in the small intestine. When fat reaches the small intestine, it is normally in the form of globules, emulsified with the rest of the alimentary components and covered by proteins and phospholipids. The size of the fat globules depends on numerous factors such as the type of fat, the presence of other substances, the mixing time, etc.

In the duodenum there is an increase in pH due to the release of bicarbonate and bile salts and this is also where the pancreatic enzymes are added. Pancreatic lipase is an enzyme that acts at the fat/water interface [2]. When the enzyme approaches an apolar medium, its tertiary structure changes and the active center is exposed. This dependence on the existence of interfaces to show activity means that aspects such as the size and surface topology of particles supplemented in the diet and also their coating could have a great influence on the enzymatic activity.

The ability of enzymes to interact with their substrates can impact the digestibility of lipids and therefore their absorption. Fat hydrolysis is a key factor in relevant diseases, such as obesity problems, one of the main pandemics in the developed world, and nutritional deficiencies such as those observed in cystic fibrosis when the enzyme supply is deficient [3]. Although, nature focuses on promoting hydrolysis to achieve the maximum use of food, the access of enzymes to substrates can be controlled by creating physical barriers in the food–enzyme system by encapsulating the fats [4]. By contrast, other strategies can be developed to enhance or modulate the digestion of fats.

The development of technology at the nanometric level has led to a revolution in very diverse fields, not the least of which is health. Nanomedicine has had a rapid and broad impact on health, with close to 100 “nanomedicines” approved or undergoing clinical trials in the period 2016–2019 [5]. Numerous developments have been achieved, encompassing in vitro detection, diagnosis, multimodal imaging, controlled release, chemically based, photochemical, genetic or immunoassay therapies or theragnostic (combination of treatment and diagnosis) developments based on a wide variety of nanomaterials [6,7]. To be sure, not only have the supports contributed to this variety, but also the surface modification chemistry that has allowed the creation of hybrid materials with a myriad of new functionalities and biological significance.

The influence of size, shape and surface nature/composition on the functioning of nanomaterials at the cellular level and in some pathologies such as cancer has been widely studied and much progress has been made in relation to toxicity, bioaccumulation, targeting, activation, etc. [8,9]. In contrast, knowledge of their behavior in other areas is much more limited. As an example, the authors have studied the influence of the topology of silica mesoporous materials and their functionalization on the ability to immobilize and inhibit the Polyphenol Oxidase enzyme (from the tyrosinase family). This enzyme is present in fruits and generates enzymatic browning, which has a great impact in the food industry [10,11]. We have observed that those materials that have pores larger than 3 nm can produce a rapid and significant immobilization of the protein. This immobilization is accompanied by a variation in activity, that can reach a complete inhibition in the case of materials functionalized with thiols. Thus, the interaction of biological molecules with nanomaterials is not innocent and can be used to modulate their activity [12,13].

In the case of nanomaterials to be used in digestive processes, the main interest of the pharmaceutical industry has focused on their use as encapsulation systems for the release of drugs and improving fat hydrolysis processes that allow maximization of the bioavailability of fat soluble drugs. The modulation of digestive enzymes has been a relatively poorly studied field, except in the fight against obesity through, for example, the inhibition of lipase to decrease the digestibility of fats. An example is the commercially available drug Orlistat, which induces an irreversible inactivation of the lipase enzyme [14].

The interaction of nanomaterials with enzymes has been widely studied because they are a fundamental element of the biotechnology industry. There is extensive knowledge of strategies for enzyme immobilization. Depending on the specific enzyme-support selected interaction, the enzymes maintain or even improve their activity, stability and selectivity [15,16]. On the other hand, the influence of mesoporous materials on lipolysis when they are mixed with fats has also been studied. When aerosil or ludox are tested in presence of fat globules and a surfactant (lecithin), the silica particles locate at the interface between water and fat, coating the fat globule. This type of behavior produces a much faster and more complete hydrolysis of fats than in model systems in which the nanomaterial is not present [17]. The same authors also verified that the size of the particles, their topology, the material to fat ratio and the surface charge are factors that modify the surface properties and therefore the ability of the lipase to access the substrate and vary the hydrolysis rate [18].

The idea of an “intelligent” modulation of lipase has been explored by Chen and colleagues [19] by using a polymeric material that was designed to be degraded by lipase releasing orlistat. At low concentrations of lipase, the material does not release orlistat, which does not interfere with the hydrolysis of the fat. However, if the enzyme concentration increases, the polymer is hydrolyzed releasing orlistat, which in turn deactivates the enzyme. This type of system allows obesity to be controlled in a more personalized way than the direct administration of orlistat. It maintains a minimum of enzymatic activity and does not incur an excess or deficiency of medication. Tests carried out with mice showed that they suffered a significant weight loss but also that the polymer is absorbed and metabolized by the body.

Thus, silica hybrid nanomaterials have great potential to be the central element of a new family of products with the ability to modulate digestive activity. However, as far as we known, no studies have been done to study the influence of functionalized silica materials with diverse porous systems added to the digestion medium, instead of using materials previously loaded with fat. The present work explores the capacity of different supports and functionalities to modulate the hydrolysis of fats under duodenal conditions.

## 2. Materials and Methods 

### 2.1. Chemicals

Tetraethyl orthosilicate (TEOS), triethanolamine (TEAH_3_), hexadecyltrimethylammonium bromide (CTAB), triethoxy(methyl)silane (TEMS), chlorotrimethylsilane (CTMS), trimethoxy(propyl)silane (TMPS), triethoxy(octyl)silane (TEOCS), pancreatic porcine lipase (L3126, Type II, 100–500 units/mg protein using olive oil with 30 min incubation, 30–90 units/mg protein using triacetin), tributiryne, tris(hydroximethyl)amino methane (TRIS) and sodium taurodeoxycholate were purchased from Sigma Aldrich (Madrid, Spain). Sodium chloride was purchased from AppliChem Panreac (Barcelona, Spain). Calcium chloride was acquired from Merck (Madrid, Spain). All the reagents were used without further purification.

### 2.2. Synthesis of the Silica Nanomaterials

UVM-7 was prepared following the atrane route. An amount of 44 mL of TEOS was mixed with 100 mL of TEAH_3_ and heated up to 140 °C. The reaction was allowed to cool, adding 18.7 g of CTAB at 120 °C and 320 mL of water at 80 °C. A white suspension was formed and allowed to age for 24 h at room temperature. The solid was filtered, washed with distilled water, dried in the oven at 50 °C for one day and calcined at 550 °C to remove the surfactant.

Massive Stöber nanoparticles (SNP). An amount of 286 mL of water, 680 mL of ethanol and finally 33 mL of ammonia were added to a plastic beaker. The mixture was left stirring for 10 min and then the TEOS was added and stirred at room temperature for 24 h. Afterwards, the material was separated by centrifugation, washed with ethanol twice and allowed to dry in the oven (Gallur, Valencia, Spain) at 70 °C for 24 h.

Mesoporous Stöber nanoparticles (MSNP) were prepared using the atrane route. An amount of 22 mL of TEOS were mixed with 50 mL of TEAH_3_ and heated up to 140 °C. The reaction was allowed to cool, adding 18,7 g of CTAB at 120 °C and a mixture of 500 mL of ethanol and 900 mL of water at 70 °C. A white suspension was formed and allowed to age for 24 h at room temperature. The solid was removed by centrifugation (7500 rpm), washed with water and ethanol twice, dried in the oven at 50 °C for 24 h and calcined at 550 °C for surfactant evolution.

### 2.3. Surface Functionalization of the Materials

The silica based materials were functionalized with TEMS, CTMS, TMPS or TEOCS. In all cases the procedure was executed as follows: 1 g of silica material (UVM-7, SNP or MSNP) was added to a solution of 10 mmol of silane (TEMS, CTMS, TMPS or TEOCS) in 30 mL of acetonitrile and allowed to react at room temperature for 24 h. Afterwards, the solid was removed by centrifugation, washed with acetonitrile and dried in the oven at 40 °C for one day.

### 2.4. Characterization of the Materials

The characterization of the tested materials was carried out by low-angle X-ray powder diffraction (XRD) (Bruker D8 Advance CuKα radiation (Madison, USA), transmission electron microscopy (TEM, JEOL-jem-1010, Peabody, USA), nitrogen adsorption/desorption isotherms and thermogravimetric analysis (TGA). Nitrogen adsorption/desorption isotherms were carried out in a Micromeritics ASAP 2010 (Norcross, USA) automated sorption analyzer at the liquid nitrogen temperature (−196 °C.). Samples were degassed at 120 °C in a vacuum overnight. The specific surface area was determined by applying the Brunauer–Emmett–Teller (BET model) [20] from the adsorption data within the low-pressure range. Pore size was calculated following the Barret–Joyner–Halenda model (BJH) [21]. From the XRD analysis [22], the a_0_ cell parameter and the wall thickness of the silica materials were calculated (see Table 1). The TGA was used to estimate the amount of the different functionalized chemical groups on the silica material surface using an oxidant atmosphere (air, 80 mL/min) with an identical heating program for all samples (10 °C/min from 393 to 1273 K and an isothermal heating step at this temperature for 30 min). 

### 2.5. Studies of Influence of the Materials Under In Vitro Digestion

The influence of the materials in the lipolysis under in vitro digestion process was measured using lipase assay protocols using the pH-stat method created by the INFOGEST Network—WG4 Lipases and lipid digestion [23]. This method is based on the assessment of the amount of sodium hydroxide necessary to neutralize the carboxylic acids generated during the hydrolysis of fats. All the measurements were performed by triplicate using the Metrohm Tiamo 902 Titrando instrument. 

The assay solution was composed by TRIS 0.3 mM, NaCl 150 mM, CaCl_2_ 2 mM, sodium taurodeoxycholate (NaTC) 4 mM in MiliQ water. To perform the assay, 14.5 mL of the assay solution was mixed with 0.5 mL of tributyrin and 10 mg of material (if necessary). The system was thermostated to 37 °C and the pH set at 8.0. An amount of 100 μL of the enzyme stock solution (1 mg lipase/mL) was added and the consumption rate of NaOH measured over 5 min. The activity of the enzyme in solution (A_s_) (U/mL) and the relative activity in the presence of the material were calculated with the equations (1) and (2), respectively. x being the μmole of NaOH per minute, v the μL of the enzyme solution added in the pH-stat vessel, A_mat_ the value of A_s_ in presence of the material and A_cont_ the value of A_s_ in absence of the material.

A_S_ = (x × 1000)/v, (1)

Relative activity (%) = 100 × A_mat_/A_cont_, (2)

### 2.6. Data Analysis

Data are reported as means ± standard deviation. Partial least-squares regression studies (PLS) were carried out with the R 3.6.0 software using the Kernel algorithm. Scale and center were used as parameters to build the model. Leave one out (LOO) cross-validation was used to evaluate the adequacy of the experimental data. The number of latent variables was selected to minimize the root mean square error of prediction (RMSEP).

## 3. Results and Discussion

### 3.1. Description of the Materials

A total of 15 materials (three supports × five functionalizations) were prepared and characterized. The supports show a wide variation in their properties to study the effect of the topology/morphology in the interaction with the fat/lipase system. The first material, SNP, was prepared using the Stöber method. It is prepared in absence of surfactant, thus massive spheres in the nanometric range were obtained. The second support, MSNP, is similar to SNP but presents porosity in the mesoporous range due to the presence of surfactant micelles in the reaction medium. Its preparation combines the Stöber and atrane methods. Finally, the UVM-7 silica shows a morphology that can be described by micrometric aggregates of nanometric mesoporous particles. This solid has bimodal porosity: intra-particle mesopores due to the templating effect of the micelles (diameter typically around 2 nm) and inter-particle macropores formed through the particle aggregation (diameter typically around 40 nm) [24].

For the functionalization, hydrophobic groups with diverse chain lengths were selected. They include methyl (C_1_), propyl (C_3_) and octyl (C_8_) functional groups, and a trimethyl silyl group (3C_1_) with strong hydrophobic properties. The fat has a hydrophobic character and the enzyme must interact with apolar substances to develop its activity. Thus, silica materials with hydrophobic groups were designed to interact with both components. Moreover, the materials without surface functionalization (with silanol groups at the surface and consequently showing a more hydrophilic character), were tested.

**Figure 1:**
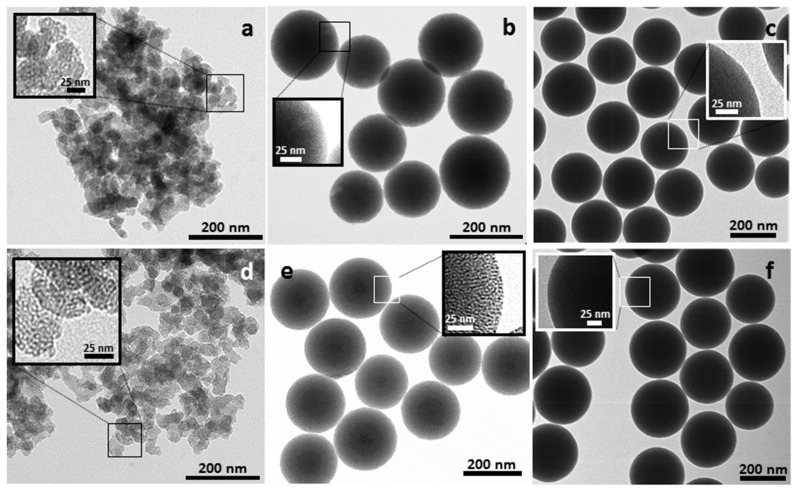
TEM images of the pure silica based materials (**a**) UVM-7, (**b**) mesoporous Stöber nanoparticles (MSNP) and (**c**) massive Stöber nanoparticles (SNP), and selected modified materials functionalized with chlorotrimethylsilane (**d**) U7-3C_1_, (**e**) MS-3C_1_ and (**f**) SP-3C_1_. Images with higher magnification are shown in the insets showing the porous (U7, U7-3C_1_, MS and MS-3C_1_) or massive (SP and SP-3C_1_) nature of the materials.

Initially, the topology of the prepared materials was checked through TEM (Figure 1). The nanoparticulate structure of UVM-7 and its tendency to form aggregates at micrometric scale and the spherical morphology of Stöber particles (both SNP and MSNP) can be appreciated. In the case of the latter particles, it is observed that they have similar average diameters in the 100–200 nm range. TEM images of U7 and MSNP (at the sphere border) materials show the characteristic white spots associated with the surfactant-generated mesopores. Obviously, these typical features are not observed in SP nonporous spheres. Upon functionalization, regardless the functional group attached or its proportion, the materials’ morphologies do not show significant differences, indicating that the basic structure of the silica supports is maintained after the functionalization. This can be viewed in the representative images included in Figure 1d–f prepared with the same functional group.

A summary of the main characteristics of the materials obtained in the characterization can be found in Table 1. Low angle XRD confirms the presence of the mesoporous ordered structure for UVM-7 and MSNP with a peak at 2θ around 2° corresponding to the reticular plane 100. Such a peak is maintained during the functionalization, indicating that the mesoporosos structure is not affected by the surface modification procedure. 

Thermogravimetric analysis shows that the value of milimoles of silane per gram of silica varies between 0.28 and 3.32. As can be seen in Table 1, the number of organic molecules attached to the material is affected by the nature of the support and the organic functional group. In general, the degree of functionalization is higher for UVM-7 than for the Stöber nanoparticles. This tendency can be understood taking into account the nanometric size of the primary UVM-7 nanoparticles that consequently offers a high “external” surface for functionalization when compared to SNP and MSNP. Furthermore, the organic content is similar for the porous (MSNP) and non-porous (SNP) nanoparticles. These results suggest that the functionalization is produced mainly in the external surface of the particles, probably due to the low diffusion available in the small size mesoporous system. In the case of the UVM-7 the higher organic content can be assigned to the presence of the larger sized textural pores. Among the silanes, as the size of the organic chain increases the number of moles reduces (C_1_ > C_3_ > C_8_), probably due to steric effects that limit the diffusion of the silanes and the access to the silica surface. In fact the number of moles added to the silica supports in the case of CTMS, a silane with three methyl groups, is similar to the propyl functionalized ones.

Adsorption-desorption isotherms were measured on the porous UVM-7 and MSNP. In the case of U7, the isotherms illustrate the presence of a bimodal pore system through the appearance of two adsorption steps in agreement with the presence of a bimodal porous system composed of mesopores and textural porosity. By contrast, MSNP only show one step, corresponding to the filling of the material’s mesopores. In both cases a surface over 1100 m^2^ g^−1^ and a mesoporous diameter close to 2.7 nm is found. U7 additionally has the textural pores with average diameters in the 30–40 nm range.

In both cases (U7 and MSNP) a decrease in the specific surface area and pore diameter is observed when the material is functionalized, as expected due to the occupancy of part of the voids by the organic groups located in a preferential way at the external surface or in the mesopore entrances. A decreasing trend of the pore size with the chain length of the organic in the size of the functional groups is not observed. This can be explained taking into account that, according to the thermogravimetric analysis, the percentage of functionalization decreases with increasing chain size of the functional groups. Thus, even though the U7-C_8_ material has a more voluminous functional group than U7-C_3_, its percentage of functionalization is lower, and similar values of specific surface are measured. The UVM-7 textural pore suffers a relatively greater decrease in the volume and size in comparison to the mesopores, suggesting that the functionalization occurs at the macropores of the material to a greater extent.

### 3.2. Effect of the Materials in the Fat Hydrolysis 

As noted above, the hydrolysis of fats is a non-trivial process based on interfacial interactions and the presence of the silica-based materials adds an additional level of complexity. Thus, for the characterization of the effect of the materials in the digestion of fats, the fat hydrolysis was measured simulating duodenal conditions using the pH-stat method (Table S1). This technique quantifies the fat hydrolysis measuring the amount of sodium hydroxide necessary to neutralize the carboxylic acids generated during the hydrolysis.

**Figure 2:**
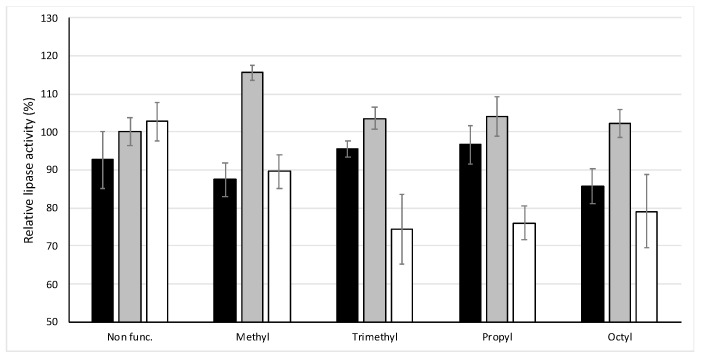
Lipase activity in the presence of bile salt (NaTC). Black column U-Cx; Grey column MS-Cx and white column SP-Cx.

Figure 2 shows how the morphology of the materials, as well as the functionalization, makes the enzymatic activity vary. In the case of non-functionalized materials, they seem to cause minor changes in lipase activity. In any case, we observed that the enzymatic activity is slightly lower when the UVM-7 silica is present. It was observed that UVM-7 functionalized materials have a tendency to decrease lipase activity, inhibiting it up to 15% when functionalized with TEMS and TEOCS. The same trend is observed for the functionalized massive Stöber nanoparticles; however, the inhibition is greater, reaching up to 25% for the materials SP-3C_1_, SP-C_3_ and SP-C_8_. On the contrary, Stöber particles with mesopores have a tendency to increase the activity of the enzyme, reaching 115% activity for MS-C_1_.

**Figure 3:**
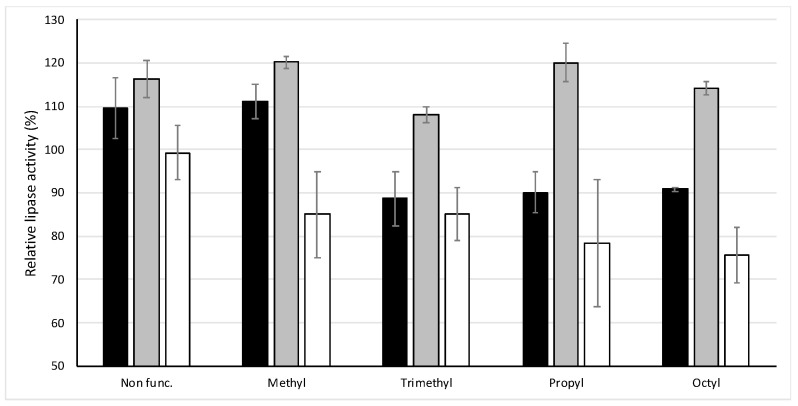
Lipase activity in the absence of bile salt (NaTC). Black column U-Cx; Grey column MS-Cx and white column SP-Cx.

Bile salts, such as NaTC, are secreted by the gall bladder and the absence of bile salts can lead to digestive disorders. They enhance colloidal stability of lipid droplets and solubilize the digestion products in water, being fundamental for the digestion and epithelial absorption of free fatty acids in the intestine [25]. Thus, due to the relevance of bile salts and the possibility that the nanomaterials cover some of their functions, the same test was carried out in the absence of the bile salt NaTC (see Figure 3). 

A first effect is that U7 and MSNP increase the speed of the reaction in comparison with the control (in absence of material and NaTC). In the case of UVM-7 based materials, the effect depends on functionalization. For U7-3C_1_, U7-C_3_ and U7-C_8_ the inhibition is maintained at 10%, however, U7-C_1_ induces an activation of 10%. In the case of the materials that contain, as support, mesoporous Stöber particles, the tendency to increase the hydrolysis kinetics is enhanced in the absence of the bile salt, reaching up to a 120% rate of hydrolysis (MS-C_1_ and MS-C_3_). SNP and its alkyl functionalized derivatives show a trend similar to that found in the presence of NaTC. 

Additionally, to study whether it was possible to achieve a greater effect on lipase, the same study was carried out in the absence of the bile salts but this time tripling the concentration of material in the solution to 30 mg for U7, MS and SP and their methyl functionalized materials U7-C_1_, MS-C_1_ and SP-C_1_. Only two materials offered significant differences when the amount of material was increased. In comparison with the assay developed in presence of 10 mg of silica material, U7-C_1_ causes a decrease in the enzymatic activity, approaching the results of the tests with NaTC. By contrast, for MS the activity further increases, reaching 140% of relative lipase activity. 

It seems that the presence of mesoporous silica nanomaterials influences the enzymatic activity of lipase. However, the complexity of the system hinders an easy explanation of the influence of the materials topology and their functionalization in the fat hydrolysis. Therefore, a more in-depth analysis of the data was carried out to gain insight into their behavior.

### 3.3. Modelization of the Parameters Influencing the Activity of the Materials 

Multivariate analysis techniques can be useful to extract valuable information on the influence of the properties and structural parameters of molecules and materials on supramolecular processes [26]. For the analysis we employed the data contained in Table 1 together with other structural parameters such as the alkyl chain length. A list of the full set of labels (11 labels) can be found in Table 2. In the case of the surface area of the massive Stöber nanoparticles, the area was estimated from the particle diameter measured by TEM following a reported procedure [27]. 

Although there are several strategies that can be used, in our case we selected Partial Least Squares Regression (PLSR). The PLS is a multivariate projection method that models the relation between an array of dependent variables (Y) and another array of independent variables (X) to find the components that allow the highest correlation with Y. In our case, the dependent variable was relative speed of fat hydrolysis (RFH) and the independent variables the rest of labels included in Table 2. In PLS, the first principal component (PC1) contains the highest explained variance and explains most of the inhibitory response; PC2 contains the second-highest explained variance, and so on.

As we were interested in including all the information from the system to gain insight about the influence of the labels in the activity, and there was a reduced number of samples (30, 5 supports × 5 functionalities × 2 with and without taurodeoxycholate), all the samples were included in the model and Leave One Out approximation used for the validation of the regression. Two latent variables were selected which contain 73% of the RFH explained variance. A list of the squared value of the loadings can be found in the Table 3. 

The contribution of the diverse labels to the principal components can be used to gain insight about the influence of each representative chemical structure parameter in the fat hydrolysis. In this case, PC1 that represents the highest explained activity, is mainly composed of the mesoporous system and the surface area, all in a positive sense. The mesoporous system would favor the fat hydrolysis, and the effect would be more pronounced when the surface or pore size and volume increases. On the other hand PC2 is mainly related with the textural pore system and the functionalization (NCA). All the components in PC2 have loadings with negative values, indicating that the presence of the textural pore system or the functionalization with long alkyl chains induces the inhibition of the lipase enzyme. Moreover, the taurodeoxycholate has a relevant contribution to PC2 suggesting that the materials are more effective in improving the relative fat hydrolysis rate in absence of NaTC. Other labels related with the functionalization, such as the total organic content (TGA), are included in the PC3, indicating that they offer only a minor contribution when all the materials are analyzed simultaneously. 

Since the three supports (U7, MS y SP) could offer diverse mechanisms of action, the PLSR was applied also to each support individually (Table 3). For the simplest material, the massive Stöber nanoparticles (SP-Cx), two latent variables were selected and the model offers a moderate fit (R^2^ = 0.710). PC1 contains the three parameters related with the surface functionalization (chain length of the alkyl unit (CLA), NCA and TGA). All of them have a negative sign indicating that higher degrees of functionalization and longer alkyl chains induce an inhibition of the lipase activity rate. Second, the complexity of the system was increased though the inclusion of mesopores (MS-Cx). For the MS materials, a model with three latent variables and R^2^ = 0.857 was obtained. By contrast with the SP particles, as the functionalization of the mesopores (TGA) increases the hydrolysis rate is powered. RFH is also favored with long chain hydrocarbons (NCA) and wider pore diameter. By contrast, higher surfaces or pore volumes inhibit the lipase activity. Finally, no proper fit was obtained for the UVM-7; a maximum of R^2^ = 0.465 was calculated with four PCs. Probably this is due to the higher heterogeneity in the particle size and conformation in comparison with the other two materials. However, if we analyze the coefficients for four components, the lipase activity improves with SUR and the mesopore size and decreases with NCA and the textural pore diameter.

From the data analysis, we can deduce that the functionalization of surfaces with alkyl chains improves the interaction of nanomaterials with the fat-lipase-NaTC system. The fat hydrolysis rate is improved by the presence of long chain hydrocarbons and mesopores, but is inhibited for flat surface nanoparticles and textural pores. This suggests that the activity of these MSNP materials could have some similarities with the bile salts, maybe by capturing the free fatty acids in the organic modified mesopore entrances avoiding the inhibition of the enzyme [28]. We cannot discard either that the mesopores increase the exposed fat surface and the hydrolysis rate. 

In contrast, the combination of mesopores and textural pores could hinder the diffusion of the hydrolysis products to the medium, inhibiting the hydrolysis. Moreover, the material could capture the lipase in a non active conformation, reducing the activity in the textural pores. According to the bibliography [29], there could be a certain adsorption of lipase in mesopores larger than 10 nm, such as the large textural pores of U7. 

Flat surface particles, such as SP materials, would offer a diverse mechanism of action; the fact that the activity is not influenced by the presence or absence of NaTC suggests that these materials can play a principal role as interfacial interference species, blocking the access of the lipase enzyme. The lipid-in-water interface is coated with SNP, restricting lipase access [29]. In presence of long alkyl chains the interaction of the material with the fat globule and, therefore, the inhibition would be stronger.

Thus, both the textural structure of the materials and their surface functionalization are key factors in the design of materials devoted to modulate the fat hydrolysis under duodenal conditions. Although further research is necessary before a full understanding of the effect of the materials in the digestion of fats, these results open the door to the preparation of new families of tailored alkyl functionalized nanomaterials to combat diseases such as obesity or cystic fibrosis. 

## Figures and Tables

**Table 1:** Textural properties and organic content of the silica based materials.

Material	Support	Functional Group	Area ^1^ (m^2^ g^−1^)	Mesopore Volume ^2^ (cm^3^ g^−1^)	Mesopore Diameter ^2^ (nm)	Textural Pore Diameter ^2^ (nm)	Textural Pore Volume ^2^ (cm^3^ g^−1^)	mmol /g SiO_2_	d_100_^3^ (Å)	2θ ^4^ (^0^)	a_0_^5^ (Å)	Dw ^6^ (Å)
U7	UVM-7	-	1146	0.98	2.78	40.59	1.60	-	40.8	2.16	47.1	19.3
U7-C_1_	UVM-7	Methyl	992	0.74	2.61	28.91	0.77	3.32	40.0	2.20	46.2	20.1
U7-C_3_	UVM-7	Propyl	1028	0.77	2.63	40.10	1.35	1.05	40.0	2.20	46.2	19.9
U7-C_8_	UVM-7	Octyl	974	0.82	2.74	31.69	0.82	0.47	43.2	2.04	49.9	22.5
U7-3C_1_	UVM-7	Trimethyl	865	0.52	2.28	29.68	0.75	1.82	43.7	2.02	50.4	27.6
SP	SNP	-	-	-	-	-	-	-	-	-	-	-
SP-C_1_	SNP	Methyl	-	-	-	-	-	2.09	-	-	-	-
SP-C_3_	SNP	Propyl	-	-	-	-	-	1.13	-	-	-	-
SP-C_8_	SNP	Octyl	-	-	-	-	-	0.32	-	-	-	-
SP-3C_1_	SNP	Trimethyl	-	-	-	-	-	1.34	-	-	-	-
MS	MSNP	-	1231	0.94	2.88	-	-	-	35.7	2.47	41.3	12.5
MS-C_1_	MSNP	Methyl	1088	0.90	2.67	-	-	1.95	36.6	2.41	42.3	15.6
MS-C_3_	MSNP	Propyl	1198	0.78	2.48	-	-	0.93	34.9	2.53	40.3	15.5
MS-C_8_	MSNP	Octyl	1136	0.77	2.53	-	-	0.28	35.2	2.51	40.6	15.3
MS-3C_1_	MSNP	Trimethyl	1190	0.83	2.15	-	-	1.27	37.0	2.39	42.7	21.2

^1^ Brunauer-Emmett-Teller (BET) specific surface calculated from the N_2_ adsorption-desorption isotherms. ^2^ Pore volumes and pore size (diameter) calculated from the N_2_ adsorption-desorption isotherms. ^3^ Diffraction peak of the reticular plane 100 calculated by 2 × d_100_ senθ = nλ ^4^ Angle of incidence for the reflection plane. ^5^ The cell parameter calculated by a_0_ = 2 × d_100_× ($\sqrt{3})$^−1^. ^6^ Wall thickness was calculated by dw = a_0__−_d_p_, where d_p_ is the pore diameter.

**Table 2:** List of labels used in the Partial Least Squares Regression (PLSR) model.

Label	Support
RFH	Relative fat hydrolysis speed (control = 100)
CLA	Chain length of the alkyl unit
NCA	Total number of carbon atoms in the alkyl unit
TGA	Organic loading determined by TGA (mmol gr SiO_2_^−1^)
SUR	Surface area (m^2^ g^−1^)
MEV	Mesopore volume (cm^3^ g^−1^)
MED	Mesopore diameter (nm)
TEV	Textural pore volume (cm^3^ g^−1^)
TED	Textural pore diameter (nm)
TC	Presence of NaTC (1 Yes/0 No)
XRD	Value of 2θ (^0^)

**Table 3:** Squared values of the loadings of the first Principal Components.

Samples	Component	CLA	NCA	TGA	SUR	MEV	MED	TEV	TED	TC	XRD
Full set of samples (30)	PC1	-	-	-	0.237	0.255	0.195	-	-	-	0.237
	PC2	-	0.055 *	-	-	-	-	0.385 *	0.419 *	0.075 *	-
SP-C_x_	PC1	0.380 *	0.515 *	0.105	-	-	-	-	-	-	-
	PC2	0.122	-	0.863 *	-	-	0.220	-	-	-	-
MS-C_x_	PC1	-	-	0.149	0.161 *		0.054	-	-	0.564 *	
	PC2	0.175	0.102	-		0.191 *		-	-	0.113 *	0.377
	PC3	0.159 *	0.280 *	-	0.233		0.054	-	-	-	0.273

Values lower than 0.05 were omitted. * Negative values were obtained for the loadings.

## Supplementary Materials

The following are available online at https://www.mdpi.com/2079-4991/10/10/1927/s1, Table S1: Lipase activity for the tested materials in presence and absence of tauro deoxycholate (NaTC).
