# Supplementary material for: Use of Silica Based Materials as Modulators of the Lipase Catalyzed Hydrolysis of Fats under Simulated Duodenal Conditions
Source: Nanomaterials (Basel). 2020 Sep 27;10(10):1927. doi: 10.3390/nano10101927 (PMC7601910; doi:10.3390/nano10101927)
Supplement: Supplementary file 1 [file nanomaterials-10-01927-s001.pdf]

Use of Silica Based Materials as Modulators of the Lipase Catalyzed Hydrolysis of Fats under Simulated Duodenal Conditions

Sara Muñoz-Pina [1], Pedro Amorós [2], Jamal El Haskouri [2], Ana Andrés [3] and José V. Ros-Lis [1,*]

[1] Inorganic Chemistry Department, REDOLí Group, Universitat de València, Burjassot, 46100 Valencia, Spain; sara.munoz@uv.es

[2] Instituto de Ciencia de Materiales, Universitat de València, C/ Catedrático José Beltrán 2, 46980 Paterna Valencia, Spain; pedro.amoros@uv.es (P.A.), Jamal.Haskouri@uv.es (J.E.H.)

[3] Instituto Universitario de Ingeniería de Alimentos para el Desarrollo (IUIAD-UPV). Universitat Politècnica de València Camino de Vera s/n, 46022 Valencia, Spain. aandres@upv.es

* Correspondence: J.Vicente.Ros@uv.es

Table S1: Lipase activity for the tested materials in presence and absence of tauro deoxycholate (NaTC)

	With NaTC			Without NaTC		
	With material U/mL	Without material U/mL	Rel. Act. %	With material U/mL	Without material U/mL	Rel. Act. %
**U7**	39,6	42,8	92,7	61,5	56,2	109,6
**U7-C_1**	53,7	61,4	87,4	49,1	44,2	111,1
**U7-C_3**	44,6	46,2	96,6	54,8	60,8	90,1
**U7-C_8**	39,2	45,7	85,8	45,0	49,6	90,8
**U7-3C_1**	35,9	37,6	95,5	52,6	59,4	88,6
**SP**	33,7	32,8	102,7	35,9	36,1	99,3
**SP-C_1**	31,1	34,8	89,5	24,8	29,1	85,0
**SP-C_3**	26,7	35,2	76,0	30,9	39,4	78,4
**SP-C_8**	23,5	29,8	79,1	23,7	31,3	75,5
**SP-3C_1**	28,6	38,5	74,3	27,5	32,3	85,1
**MS**	45,7	45,6	100,2	71,2	61,2	116,4
**MS-C_1**	55,1	47,7	115,6	63,0	52,4	120,2
**MS-C_3**	52,9	50,9	104,1	71,1	59,2	120,0
**Ms-C_8**	53,2	52,0	102,3	60,4	53,0	114,1
**MS-3C_1**	48,9	47,2	103,6	52,8	48,9	108,0
